# Urinary pesticide profiles and liver disease risk in Thailand: a machine-learning risk-prediction model

**DOI:** 10.1101/2025.09.19.25336162

**Published:** 2025-09-22

**Authors:** Daxesh P. Patel, Christopher A. Loffredo, Majda Haznadar, Mohammed Khan, Amelia L. Parker, Benjarath Pupacdi, Siritida Rabibhadana, Panida Navasumrit, Nirush Lertprasertsuke, Anon Chotirosniramit, Chawalit Pairojkul, Vor Luvira, Ake Pugkhem, Wattana Sukeepaisarnjaroen, Teerapat Ungtrakul, Thaniya Sricharunrat, Kannika Phornphutkul, Frank J. Gonzalez, Anuradha Budhu, Chulabhorn Mahidol, Xin W. Wang, Mathuros Ruchirawat, Curtis C. Harris, TIGER-LC Consortium

**Affiliations:** 1 Laboratory of Human Carcinogenesis, Center for Cancer Research, National Cancer Institute, Bethesda, Maryland, USA; 2 Georgetown University Medical Center, Washington, DC, USA; 3 Translational Research Unit, Chulabhorn Research Institute, Bangkok, Thailand; 4 Laboratory of Chemical Carcinogenesis, Chulabhorn Research Institute, Bangkok, Thailand; 5 Laboratory of Environmental Toxicology, Chulabhorn Research Institute, Bangkok, Thailand; 6 Center of Excellence on Environmental Health and Toxicology (EHT), OPS, MHESI, Thailand; 7 Department of Pathology, Faculty of Medicine, Chiang Mai University, Chiang Mai, Thailand; 8 Department of Surgery, Faculty of Medicine, Chiang Mai University, Chiang Mai, Thailand; 9 Faculty of Medicine, Khon Kaen University, Khon Kaen, Thailand; 10 Princess Srisavangavadhana Faculty of Medicine, Chulabhorn Royal Academy, Bangkok, Thailand; 11 Chulabhorn Hospital, Chulabhorn Royal Academy, Bangkok, Thailand; 12 Rajavej Hospital, Chiang Mai, Thailand; 13 Cancer Innovation Laboratory, Center for Cancer Research, National Cancer Institute, National Institutes of Health, Bethesda, Maryland, USA.

**Keywords:** Pesticide exposure, Hepatocellular carcinoma, Chronic liver disease, Environmental epidemiology, Risk prediction modeling, Low- and middle-income countries

## Abstract

**Background:**

Building on evidence linking urinary glyphosate to chronic liver disease (CLD) and hepatocellular carcinoma (HCC), we developed urinary pesticide profiling integrated with machine learning risk prediction (MLRP) to stratify risk in high-exposure populations.

**Methods:**

We conducted a case–control study within the Thailand Initiative in Genomics and Expression Research for Liver Cancer (TIGER-LC; 2011–2016; n=593): 228 CLD, 116 HCC, and 249 controls. Eight urinary pesticides were quantified by LC–MS/MS (pendimethalin, oxadiazon, metsulfuron-methyl, butachlor, 2,4-dichlorophenoxyacetic acid [2,4-D], cypermethrin, flocoumafen, bromadiolone). A composite Pesticide Load Score (PLS), with and without glyphosate, estimated burden. Two predictive models were developed: a logistic-regression Pesticide-Informed Liver Cancer Risk Score (PILCRS) and an Extreme Gradient Boosting (XGBoost) classifier that incorporated age, sex, alcohol use, occupation, and PLS. Internal validity used 1,000 bootstrap resamples with optimism-corrected calibration.

**Findings:**

Predicted CLD probability increased from 30% in the lowest PLS quartile to over 70% in the highest, and HCC from 10% to 40% (p<0·0001). Relative estimates were consistent; the highest versus lowest quartile yielded odds ratios of 2·84 (95% CI 1·66–4·91) for CLD and 4·76 (2·30–10·29) for HCC. Cypermethrin remained independently associated. After optimism correction, both models demonstrated strong discrimination and calibration.

**Interpretation:**

This framework establishes a scalable, exposure-informed tool for liver disease prediction. Findings underscore pesticide burden as a modifiable risk factor and align with Sustainable Development Goal 3·9 and WHO–FAO priorities in low- and middle-income countries (LMICs). External validation is essential.

**Funding:**

National Institutes of Health (USA); Thailand Science Research and Innovation.

## Introduction

Pesticide exposure is an escalating planetary health and environmental justice concern, particularly in LMICs, where regulatory infrastructure, exposure surveillance, and mitigation strategies remain critically underdeveloped. Agricultural intensification—driven by global food demand, climate adaptation, and market liberalization—has accelerated the use of hepatotoxic agrochemicals in regions where internal exposure monitoring and occupational safeguards remain limited.^1^ The implications for liver disease are substantial, given that pesticide-induced oxidative stress, mitochondrial dysfunction, and DNA damage are established pathways of hepatic injury and carcinogenesis.^2–5^

Widely used herbicides such as glyphosate, paraquat, and 2,4-D continue to be applied extensively in agriculture and home gardens despite mechanistic evidence linking them to hepatotoxicity and liver tumourigenesis.^2,4,6–8^ Glyphosate is metabolised to aminomethylphosphonic acid (AMPA) and a phosphoric acid derivative (PPA), both detectable in urine and serving as biomarkers of internal exposure, with toxicological evidence implicating them in oxidative stress, DNA damage, and hepatotoxicity.^2,9,10^ Other commonly deployed compounds—including the herbicides pendimethalin, oxadiazon, metsulfuron-methyl, butachlor, and the insecticide cypermethrin—exhibit hepatotoxic, pro-inflammatory, and fibrogenic effects in experimental models.^2,4,6,8–19^ Second-generation anticoagulant rodenticides, such as flocoumafen and bromadiolone, though less studied in humans, are environmentally persistent and induce hepatic injury via oxidative stress and coagulopathy.^6,12,19^ These compounds also contribute to groundwater contamination and biodiversity loss, compounding their ecological impact.^1,6^

Despite strong toxicological evidence of hepatotoxicity, epidemiological studies linking pesticide exposure to CLD and HCC remain scarce, often relying on indirect proxies prone to exposure misclassification and residual confounding.^2,19^ Real-world exposure typically involves chronic, low-dose contact with multiple compounds, patterns rarely captured by conventional assessment tools. The absence of internal dose surveillance and weak regulatory enforcement in LMICs further obscures population-level risk.^15,17^

Thailand exemplifies this dual burden. The country reports among the highest pesticide application rates in Southeast Asia and a rising incidence of CLD and HCC, alongside established risk factors such as chronic viral hepatitis, alcohol use, aflatoxins, and metabolic dysfunction.^1,2,12,20^ In high-intensity agricultural regions, herbicides such as glyphosate, 2,4-D, paraquat, and butachlor, and the insecticide cypermethrin, are used extensively under limited oversight.^1,2,14^ Our recent analysis from the TIGER-LC study identified significant associations between urinary exposure to glyphosate and its metabolites and increased risks of both CLD and HCC.^21^

To address these gaps, we explored a hospital-based case–control study nested within TIGER-LC. Using high-resolution LC–MS/MS, we quantified eight additional urinary pesticides and derived a composite PLS to estimate cumulative internal burden. These biospecimen-anchored metrics, combined with demographic and behavioural covariates, informed two predictive models—a logistic regression–based PILCRS and an XGBoost classifier—built and internally validated for discrimination and calibration within an MLRP framework. This interpretable, scalable framework is adaptable to artificial intelligence (AI)-enabled public-health tools for risk stratification and early prevention, aligns with WHO–FAO priorities and SDG 3·9, and underscores the need for strengthened regulation, surveillance, and policy to mitigate preventable liver-disease burden in LMICs.

## Materials and Methods

### Study Design and Participants

We conducted a secondary analysis within TIGER-LC, a multicentre, hospital-based case–control study led by the Chulabhorn Research Institute (CRI) in Bangkok and the US National Cancer Institute (NCI). Between 2011 and 2016, newly diagnosed HCC and CLD cases were recruited from five tertiary hospitals across Thailand (table 1). Hospital-based controls were recruited to approximate the age (within ±5 years), sex, and regional distribution of cases, although the groups were not fully balanced due to variations in case and control availability across sites and time. Questionnaire data and biospecimens were collected at enrollment. Detailed study design and clinical eligibility criteria have been described previously.^22,23^

### Exposure Assessment

Urinary concentrations of eight pesticides—2,4-D, pendimethalin, oxadiazon, metsulfuron-methyl, butachlor, cypermethrin, flocoumafen, and bromadiolone—were quantified using LC–MS/MS on a Waters Acquity Ultra Performance Liquid Chromatography (UPLC) system coupled to a Xevo Triple Quadrupole–Sensitive (TQ-S) micro mass spectrometer equipped with a Z-Spray^™^ electrospray ionization source, operated in both positive and negative ion modes. Calibration curves spanned 0.001–12.5 μM. Limits of detection (LOD) ranged from 0.16 to 467 nM, and limits of quantification (LOQ) from 0.5 to 467 nM. Matrix effects predominantly resulted in signal enhancement, and extraction recoveries exceeded 80% across analytes. Glyphosate and its primary metabolites—AMPA and PPA—were quantified separately using GC–MS, as previously described.^14^ Full assay procedures, instrument settings, and compound-specific transitions are detailed in the appendix (*Targeted LC–MS/MS assay for cross-sectional quantification of urinary pesticides: sample preparation, instrument parameters, and analytical performance*; appendix p 2) and summarised in (appendix table S1, appendix p 3).

### Expansion of Pesticide Panel and Rationale

Building on prior findings linking urinary glyphosate and its metabolites—AMPA and PPA—to increased liver disease risk.^22^ we expanded the biomarker panel to capture real-world, multicomponent pesticide exposure in Thai agricultural populations. Eight additional pesticides were selected based on participant-reported use,^9,20^ regional agricultural patterns and regulatory challenges,^9,14^ and mechanistic toxicology evidence involving hepatotoxicity, oxidative stress, and inflammatory injury.^12,20,22^ These included two herbicides, three insecticides, and three rodenticides, reflecting broad chemical class representation and plausible hepatic mechanisms (appendix table S1, appendix p 3).

### PLS Calculation

To quantify cumulative pesticide exposure burden, we constructed a composite pesticide load score (PLS) by integrating multiple urinary pesticide measurements. Two variants were defined: PLS_11_, incorporating 11 pesticides—pendimethalin, oxadiazon, metsulfuron-methyl, butachlor, 2,4-D, cypermethrin, flocoumafen, bromadiolone, glyphosate, and its two primary metabolites (AMPA and PPA); and PLS_8_, comprising the first eight of these pesticides without glyphosate or its metabolites.

All urinary pesticide concentrations were expressed in nanomolar (nM) or picomolar (pM) units and normalized to urinary creatinine, as assessed using the Jaffe method, to account for urine dilution.^14^ Calibration ranges, detection limits, and assay procedures are detailed in the appendix (*Targeted LC–MS/MS assay for cross-sectional quantification of urinary pesticides: sample preparation, instrument parameters, and analytical performance*; appendix p 2) and summarised in (appendix table S1, appendix p 3).

For each participant, the non-normalized PLS was calculated as the sum of individual analyte concentrations:

$$PLS_{i}^{\left(n\right)}=\sum_{j=1}^{n}C_{ij}$$

In this formulation, $PLS_{i}^{\left(n\right)}$ represents the cumulative PLS for participant i, derived from n compounds (n=11 for PLS_11_ or 8 for PLS_8_). The term $C_{ij}$ denotes the creatinine-normalised concentration of the jth pesticide in participant i, where i indexes study participants and j indexes individual analytes. This additive model captures the internal burden associated with real-world, multi-compound pesticide exposure and reflects the potential for additive or synergistic toxicological effects.^2,18,20,22,24^

To enable comparison across exposure strata, each participant’s PLS was normalized to the median score among hospital-based controls, yielding a fold-change (FC) metric:

$$PLS_{FC_{i}}^{\left(n\right)}=\frac{PLS_{i}^{\left(n\right)}}{P\tilde{L}S^{\left(n\right)}}$$

Here, $PLS_{FC_{i}}^{\left(n\right)}$ denotes the fold-change in pesticide burden for participant i, and $P\tilde{L}S^{\left(n\right)}$ is the median PLS score across all participants or a defined reference group. These normalized scores were used to stratify exposure groups in regression analyses and machine learning–based risk prediction models.^25^

### Covariates

Covariates were selected based on established or biologically plausible associations with liver disease risk and pesticide exposure. Data were collected at enrolment using structured questionnaires and clinical assessments. Included variables were age, sex, educational attainment, geographic region, self-reported agricultural occupation, body mass index (BMI), and serological status for hepatitis B virus (HBV) and hepatitis C virus (HCV). All statistically significant covariates were included in both multivariable regression and machine learning models to account for confounding and to improve predictive performance.

### MLRP framework (PILCRS & XGBoost)

We implemented a dual-framework MLRP strategy to estimate liver disease risk associated with cumulative pesticide exposure, developing two supervised classifiers: a multivariable logistic regression model to generate the PILCRS, and a non-linear ensemble model using XGBoost.^25^ Both models included age, sex, alcohol use, self-reported agricultural occupation, and internal pesticide burden as covariates. Exposure was modelled in three forms: PLS_11_, comprising glyphosate, its metabolites, and eight urinary pesticides; PLS_8_, comprising eight pesticides only; and cypermethrin concentration as a single compound. All exposure metrics were modelled as continuous variables to preserve scale fidelity and enhance interpretability.

The binary outcome was defined as the presence of CLD or HCC versus hospital-based controls. Logistic regression models were parameterized to yield interpretable coefficients.^24^ A representative model was specified as:

$$PILCRS=\beta_{0}+\beta_{1}\cdot Sex+\beta_{2}\cdot Alcohol+\beta_{3}\cdot Age+\beta_{4}\cdot Exposure+\beta_{5}\cdot Occupation$$

Logistic regression models were used to estimate associations between covariates and liver disease. Each β coefficient represents the change in the log-odds of liver disease per unit increase in its corresponding covariate, conditional on all other covariates in the model. Specifically, $\beta_{1}$ reflects the effect of sex, $\beta_{2}$ captures the effect of alcohol use, $\beta_{3}$ represents the age-associated risk, $\beta_{4}$ quantifies the contribution of pesticide exposure (PLS_11_, PLS_8_, or cypermethrin), and $\beta_{5}$ denotes the effect of agricultural occupation. The intercept ($\beta_{0}$) represents the model-predicted log-odds of liver disease when all covariates are held at their reference values and is not interpretable as an absolute clinical risk. In parallel, XGBoost models were optimized through grid search across key hyperparameters, including maximum tree depth, learning rate (η), subsampling ratio, and both L1 (Lasso) and L2 (Ridge) regularization; L1 penalizes the absolute magnitude of coefficients and promotes feature selection, whereas L2 penalizes the squared magnitude of coefficients and retains all features.^25,26^

### Internal Validation and Model Evaluation

Internal validation of the MLRP framework was conducted using 1,000 bootstrap resamples to assess model robustness, reproducibility, and potential overfitting.^26^ Model discrimination was evaluated using area under the receiver operating characteristic curve (AUC), and calibration was assessed using LOESS-smoothed calibration curves, bootstrapped calibration slope distributions, and the Hosmer–Lemeshow goodness-of-fit test.^26^ All models included age, sex, alcohol use, occupation, and internal exposure metrics (PLS_11_, PLS_8_, or cypermethrin) as covariates.

To evaluate predictor contributions and enhance model interpretability within the MLRP framework, Shapley Additive Explanations (SHAP) were applied to the XGBoost classifiers.^27,28^ SHAP values were used to rank variables by importance and to visualise marginal effects on predicted risk. Complete model coefficients, discrimination metrics, calibration slopes, and classification thresholds were calculated to support assessment of model performance and generalisability.

### Statistical Analysis

Analyses were conducted in R (version 4.5.0) and RStudio (version 2025.05.1). Continuous variables were summarized as mean ± standard deviation (SD); categorical variables as counts (%). Group differences were assessed via Kruskal–Wallis and chi-squared tests; pairwise comparisons used Dunn’s test with Bonferroni correction. Multivariable logistic regression and XGBoost were implemented as components of the MLRP framework to evaluate exposure–outcome associations. All p-values were two-sided; p < 0.05 denoted statistical significance. Analyses and data presentation adhered to Strengthening the Reporting of Observational Studies in Epidemiology (STROBE),^26^ and Transparent Reporting of a Multivariable Prediction Model for Individual Prognosis or Diagnosis (TRIPOD) guidelines.^27^

### Ethics Approval

The study was approved by the institutional review boards of all participating institutions. Written informed consent was obtained from all participants prior to enrollment.

## Results

### Study Population Characteristics

Among 593 participants, 228 had CLD, 116 had HCC, and 249 were hospital-based controls (table 1). Compared with hospital-based controls, CLD cases were younger and HCC cases older, with significant differences in sex, occupation, and HBV/HCV status (all *p*<0·001). Thai ethnicity was uniformly prevalent and did not differ significantly across groups (*p*=0·38).

### Pesticide Profiling and Exposure Patterns

**Figure 1:**
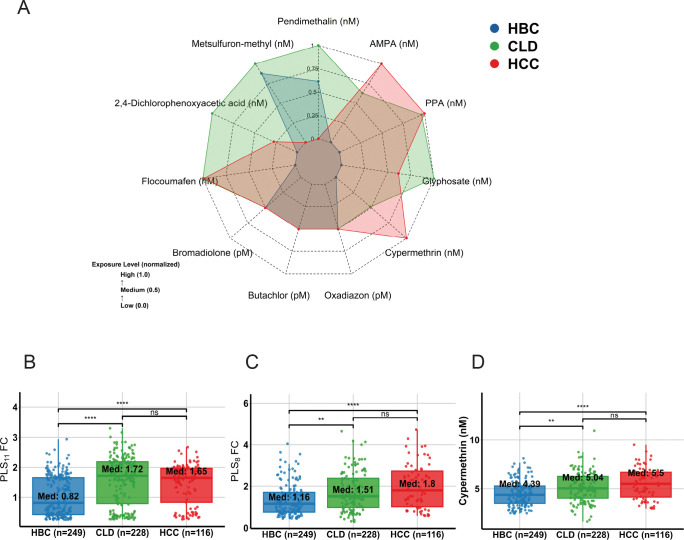
Urinary pesticide concentrations and exposure scores by disease group. (A) Radar plot shows scaled median urinary concentrations of 11 pesticides across hospital-based controls (HBC), chronic liver disease (CLD), and hepatocellular carcinoma (HCC) groups. (B–D) Boxplots display groupwise distributions of PLS_11_ fold-change (B), PLS_8_ fold-change (C), and cypermethrin (nM) (D) in HBC, CLD, and HCC groups. Asterisks denote significance by Wilcoxon rank-sum test; *ns* = not significant; Median values shown within boxes.

Urinary concentrations of 11 analytes—including glyphosate, its metabolites AMPA and PPA, and eight additional pesticides—were quantified using LC–MS/MS and GC–MS, with quality control including matrix-effect testing, recovery validation, and creatinine normalisation (appendix table S1, appendix p 3). Radar plots illustrated multidimensional exposure profiles across all analytes, with consistently higher concentrations in CLD and HCC compared with hospital-based controls (figure 1A). Individual distributions of seven additional pesticides are presented in (appendix figure S1, appendix p 5).

### Composite Exposure Metrics and Cypermethrin Stratification

Two composite indices were derived: PLS_11_, comprising glyphosate, its metabolites, and eight urinary pesticides; and PLS_8_, comprising the eight pesticides alone. Both were scaled as fold-changes relative to hospital-based control medians and were significantly elevated in CLD and HCC (figure 1B–C). Cypermethrin was analyzed separately because it dominated the exposure distribution and was markedly higher in CLD and HCC than in hospital-based controls (figure 1D).

### Multivariable Regression Analysis

Multivariable logistic regression adjusted for age, sex, alcohol use, and occupation showed that PLS_11_ and cypermethrin were significantly associated with higher odds of disease, whereas PLS_8_ showed weaker associations. For PLS_11_, adjusted ORs for the highest quartile were 2·84 (95% CI 1·66–4·91) for CLD and 4·76 (2·30–10·29) for HCC (table 2). For PLS_8_, ORs were 1·54 (0·89–2·69) for CLD and 1·76 (0·92–3·40) for HCC (table 2). For cypermethrin, ORs were 1·88 (1·04–3·46) for CLD and 3·26 (1·70–6·34) for HCC (table 2). Sensitivity analyses excluding HBV/HCV-positive participants and dose–response trends supported these findings. In fully adjusted models, high versus low PLS_11_ exposure was associated with ORs of 2·01 (95% CI 1·18–3·47; *p*=0·0115) for CLD and 1·80 (1·21–2·70; *p*=0·0042) for HCC (appendix figure S2, appendix p 6). For cypermethrin, ORs were 3·53 (1·68–7·79; *p*=0·0012) for CLD and 1·69 (0·99–2·90; *p*=0·0530) for HCC. No effect modification was observed by HBV/HCV status or occupation. Among CLD cases, males had higher PLS_11_ values than females (*p*<0·001).

### Predictive Modelling, Classifier Performance, and Predicted Risk Probability

**Figure 2:**
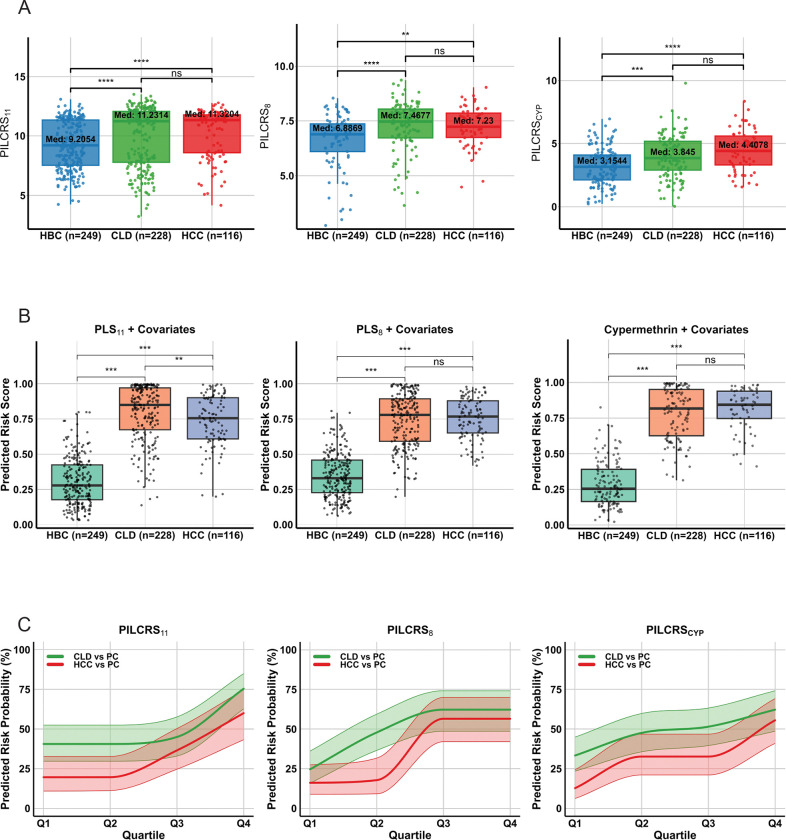
Predictive liver disease risk scores derived from PLS + covariates models by disease group. (A) Boxplots show distributions of PILCRS_11_, PILCRS_8_, and PILCRS_cyp_ across HBC, CLD, and HCC, derived from logistic regression models incorporating PLS scores or cypermethrin plus covariates. (B) Predicted liver disease risk scores from logistic regression models using PLS_11_, PLS_8_, or cypermethrin plus covariates. (C) Quartile-based predicted risk probabilities for PILCRS_11_, PILCRS_8_, and PILCRS_cyp_, showing monotone increasing estimates with 95% confidence intervals for CLD vs PC and HCC vs PC. Boxes indicate medians and IQRs; horizontal bars show Wilcoxon rank-sum test results; *ns* = not significant.

To evaluate predictive performance within the MLRP framework, three logistic regression–based PILCRS models (PILCRS_11_, PILCRS_8_, and PILCRS_CYP_) and parallel XGBoost classifiers were developed (figure 2A–B). PILCRS_11_ showed the highest discrimination among logistic models (AUC 0·85 for CLD, 0·89 for HCC), followed by PILCRS_8_ (0·83 and 0·87) and PILCRS_CYP_ (0·78 and 0·84). XGBoost using PLS scores or cypermethrin exposure plus covariates achieved AUCs of 0·86 for CLD and 0·91 for HCC, with Brier scores of 0·09 and 0·07, indicating excellent calibration. Risk score distributions showed clear separation between cases and hospital-based controls, most pronounced for PILCRS_11_ and PILCRS_CYP_ (figure 2A–B). Stratified curves demonstrated progressive increases in predicted risk probabilities with higher scores (figure 2C): for PILCRS_11_, CLD rose from 45% in Q1 to 70% in Q4 and HCC from 10% to 21%; for PILCRS_8_, CLD from 47% to 53% and HCC from 4% to 9%; and for PILCRS_CYP_, CLD from 25% to 59% and HCC from 5% to 15% (figure 2C).

### Model Calibration, Interpretation, and Internal Validation

**Figure 3:**
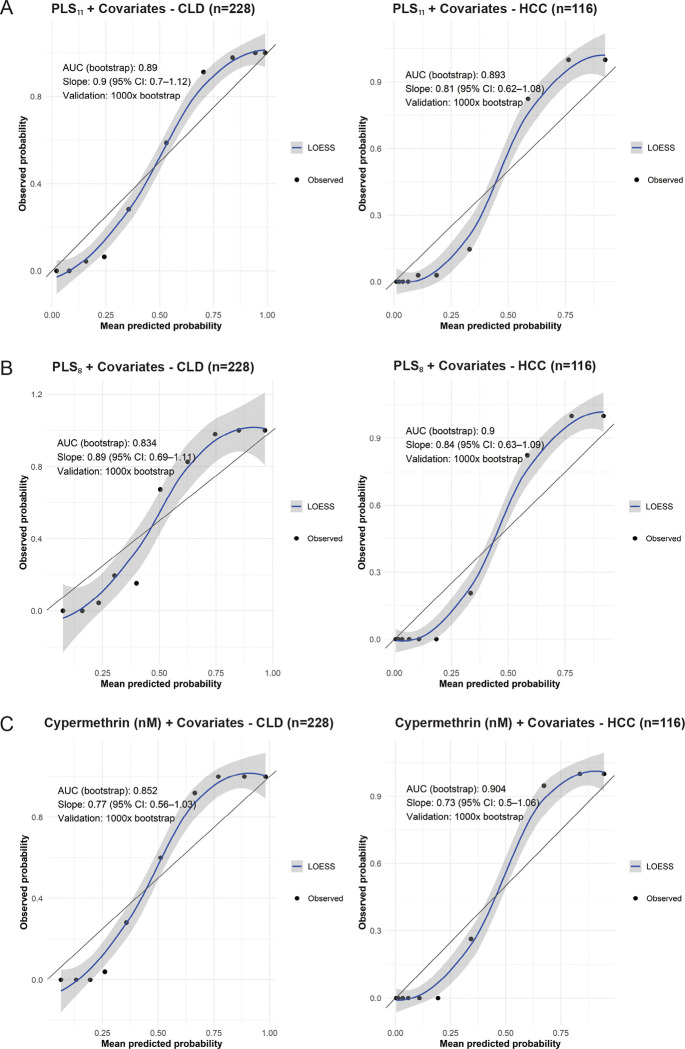
Calibration performance of PILCRS models with covariates. Calibration plots show observed versus predicted probabilities from logistic models incorporating PLS_11_ (A), PLS_8_ (B), or cypermethrin (nM) (C) plus covariates for CLD (left panels) and HCC (right panels), compared with HBC. Curves represent LOESS smoothing of observed probabilities; shaded areas show 95% CI. The black diagonal line indicates perfect calibration. AUC (bootstrap), slope (95% CI), and 1,000× bootstrap validation results are shown within each panel.

All models showed acceptable calibration, with Hosmer–Lemeshow tests non-significant (*p* > 0·10). Calibration plots from PLS plus covariates models (figure 3) showed good agreement between observed and predicted probabilities across PILCRS_11_, PILCRS_8_, and PILCRS_CYP_. Bootstrap validation (1000 resamples) confirmed robustness, with optimism-corrected AUCs of 0·83–0·90 and calibration slopes of 0·73–0·90. Distributions of slope estimates indicated minimal overfitting, and bootstrap confidence intervals supported stability (appendix figure S3, appendix p 7). Discrimination was strong, with consistent case–hospital-based control separation (appendix figure S4, appendix p 8).

PILCRS_11_ achieved the strongest performance. For CLD, it yielded an optimism-corrected AUC of 0·890 and calibration slope 0·900 (95% CI 0·705–1·118). For HCC, PILCRS_11_ (AUC 0·893; slope 0·814) and PLS_8_ (AUC 0·900; slope 0·840) both showed excellent discrimination and well-aligned calibration. Cypermethrin-based models retained predictive value (AUCs ≥0·85; slopes ≥0·70), though lower CI bounds for slope occasionally exceeded 1·0, indicating acceptable to good performance (appendix table S2, appendix p 4).

SHAP analysis decomposed predictor contributions across classifiers. In PLS_11_ models, PLS_11_ contributed most, followed by age, alcohol use, occupation, and sex. In PLS_8_ models, PLS_8_ and age were dominant, with alcohol use and occupation also important. In cypermethrin models, cypermethrin was primary, followed by alcohol use, age, occupation, and sex (appendix figure S5, appendix p 9). Contributions were directionally coherent and biologically plausible, supporting the framework’s relevance.

## Discussion

Leveraging biospecimens from the TIGER-LC hospital-based case–control study in Thailand—where pesticide exposure is extensive, monitoring infrastructure is still emerging, and HCC incidence is increasing—we found that urinary pesticide burden, particularly from PLS_11_, PLS_8_, and cypermethrin, was consistently higher in CLD and HCC cases than in hospital-based controls. Cypermethrin dominated the exposure distribution and, together with glyphosate-derived AMPA and PPA, emerged as a strong independent predictor across models.^9,14^

The magnitude of risk was substantial. Individuals with elevated PLS_11_ exposure had nearly three-fold higher odds of CLD and five-fold higher odds of HCC compared with hospital-controls, while cypermethrin exposure was associated with up to a three-fold increase.^3^ Predicted probabilities also increased across exposure strata, with CLD probability approaching 70% in the highest quartile compared with below 50% in the lowest, and HCC probability roughly doubling. Predictive models, particularly PILCRS_11_, achieved excellent discrimination and calibration,^27,28^ while gradient boosting further improved performance.^27^ SHAP analyses confirmed interpretability, with pesticides, age, and alcohol use contributing most strongly.^6^

These associations are biologically coherent and supported by mechanistic evidence. Cypermethrin induces mitochondrial dysfunction and NF-κB–mediated inflammation,^8,10,13^ while glyphosate analytes disrupt redox balance and DNA repair.^2,9,11^ Other pesticides—including pendimethalin, oxadiazon, metsulfuron-methyl, butachlor, 2,4-D, flocoumafen, and bromadiolone—exert overlapping hepatotoxic effects through oxidative stress, lipid peroxidation, and coagulation disruption.^6,7,15–17,19^ Supra-additive toxicities further justify integrative indices such as PLS_11_ for capturing cumulative burden.^2,24–26^

Thailand, one of Southeast Asia’s largest pesticide consumers, lacks national biomonitoring and does not incorporate internal exposure into prevention policy.^20,22^ In this study, urinary concentrations of cypermethrin and glyphosate analytes frequently exceeded thresholds linked to hepatic injury.^9^ If externally validated, these findings have broad implications for LMICs with similar agrochemical practices and limited regulatory infrastructure,^21^ underscoring pesticide burden as a modifiable planetary health and environmental justice risk factor, disproportionately affecting rural and agricultural workers.^1,4^ Our exposure-informed MLRP framework addresses surveillance gaps by combining biospecimen-based exposure assessment with interpretable modelling.^27^ Direct quantification minimised recall bias, while bootstrap resampling strengthened internal validity.^28^

Limitations include a hospital-based case–control design that may introduce selection bias and restrict generalisability; single-spot urine exposure assessment with potential temporal variability, urine dilution from variable hydration, left-censoring at the limit of detection, and batch effects; an expanded yet incomplete pesticide panel with some non-specific metabolites; residual confounding from hepatitis B and C, alcohol, aflatoxin, diet, and metabolic risk factors; modest HCC and CLD sample sizes limiting subgroup analyses; and MLRP models validated internally only, lacking temporal and external validation to assess overfitting, transportability, calibration, and performance across population subgroups. Future studies should prioritise prospective cohorts with repeated urine sampling and temporal anchoring, incorporate untargeted exposomic screening, and integrate host-response data—including transcriptomics, immunophenotyping, and environmental DNA—to strengthen causal inference.^25,26^ With external validation, this modular MLRP framework could evolve into AI-enabled tools for population-level risk monitoring. Urinary pesticide profiling is minimally invasive and field-deployable,^2^ supporting integration into registries, occupational health programs, and WHO–FAO frameworks.^1,4^ By advancing a harmonized model grounded in biospecimen-derived data, reproducible MLRP, and environmental health informatics, this study delivers a scalable, policy-relevant solution aligned with the WHO Global Cancer Control Strategy, the IARC Cancer Prevention Roadmap, and SDG 3·9,^3,5,29,30^ contributing to an equity-driven planetary-health model that strengthens pesticide regulation, supports early prevention, and addresses environmental injustice in LMICs.

## Supplementary Material

Supplement 1

## Figures and Tables

**Table 1:** Demographic and clinical characteristics of TIGER-LC participants by disease group

TIGER-LC participants: demographic and clinical characteristics			

Variable	Hospital-Based Controls	CLD Cases	HCC Cases

**Total N**	249	228	116
**Age (Mean ± SD)**	53·5 (9·7)	48·1 (12·3)	54·7 (10·2)
**Sex**			
Male	172 (69·1%)	120 (52·6%)	93 (80·2%)
Female	77 (30·9%)	108 (47·4%)	23 (19·8%)
**Thai ethnicity**	244 (98·0%)	227 (99·6%)	114 (98·3%)
**Longest Occupation**			
Agriculture	66 (26·5%)	30 (13·2%)	57 (49·1%)
Non-agriculture	174 (69·9%)	198 (86·8%)	57 (49·1%)
Missing Details	9 (3·6%)	0	2 (1·7%)
**HBV status**			
Positive	7 (2·8%)	185 (81·1%)	56 (48·3%)
Negative	239 (96·0%)	30 (13·2%)	46 (39·7%)
Missing Details	3 (1·2%)	13 (5·7%)	14 (12·1%)
**HCV status**			
Positive	1 (0·4%)	16 (7·0%)	22 (19·0%)
Negative	239 (96·0%)	203 (89·0%)	77 (66·4%)
Missing Details	9 (3·6%)	9 (3·9%)	17 (14·7%)

1. Tests used: ANOVA for continuous variables; χ^2^ or Fisher’s exact test for categorical variables.

2. Missing values shown as *n* (%); complete-case analysis performed.

**Table 2:** Odds ratios for chronic liver disease and hepatocellular carcinoma by quartiles of pesticide exposure metrics

Quartiles	Hospital-Based controls	CLD cases			HCC cases		

PLS_11_	n (%)	n (%)	OR (95% CI)	*p* value	n (%)	OR (95% CI)	*p* value

**Q1 (BLQ - 0·16)**	70 (28·1%)	57 (25·0%)	1·00 (Reference)	--	16 (13·8%)	1·00 (Reference)	--
**Q2 (0·16 – 0·77)**	79 (31·7%)	33 (14·5%)	0·51 (0·29 – 0·91)	0·0162	22 (19·0%)	1·22 (0·56 – 2·69)	0·7158
**Q3 (0·77 – 2·22)**	60 (24·1%)	45 (19·7%)	0·92 (0·53 – 1·60)	0·7914	34 (29·3%)	2·47 (1·19 – 5·29)	0·0121
**Q4 (2·22 – 8·74)**	40 (16·1%)	93 (40·8%)	2·84 (1·66 – 4·91)	<0·0001	44 (37·9%)	4·76 (2·30 – 10·29)	<0·0001
***p* Values for Trend**	--	--	--	<0·0001	--	--	<0·0001

**PLS_8_**							

**Q1 (BLQ - 6·90×10^−8^)**	66 (26·5%)	54 (23·7%)	1·00 (Reference)	--	29 (25·0%)	1·00 (Reference)	--
**Q2 (7·11×10^−8^ – 0·46)**	66 (26·5%)	59 (25·9%)	1·09 (0·64 – 1·86)	0·7979	23 (19·8%)	0·79 (0·39 – 1·59)	0·5153
**Q3 (0·46 – 1·78)**	68 (27·3%)	53 (23·2%)	0·95 (0·56 – 1·63)	0·8971	26 (22·4%)	0·87 (0·44 – 1·71)	0·7492
**Q4 (1·78 – 110·0)**	49 (19·7%)	62 (27·2%)	1·54 (0·89 – 2·69)	0·1146	38 (32·8%)	1·76 (0·92 – 3·40)	0·0903
***p* values for trend**	--	--	--	0·1728	--	--	0·0696

**Cypermethrin (nM)**							

**Q1 (BLQ - 12·39)**	160 (64·3%)	131 (57·5%)	1·00 (Reference)	--	61 (52·6%)	1·00 (Reference)	--
**Q2 (12·39 – 27·78)**	33 (13·3%)	29 (12·7%)	1·07 (0·59 – 1·93)	0·8884	14 (12·1%)	1·11 (0·51 – 2·31)	0·8581
**Q3 (27·78 – 63·78)**	32 (12·9%)	31 (13·6%)	1·18 (0·66 – 2·12)	0·5786	11 (9·5%)	0·90 (0·39 – 1·98)	0·8535
**Q4 (63·78 – 1943·30)**	24 (9·6%)	37 (16·2%)	1·88 (1·04 – 3·46)	0·034	30 (25·9%)	3·26 (1·70 – 6·34)	0·0002
***p* values for trend**	--	--	--	0·0394	--	--	0·0013

*n* = number of samples; OR = odds ratio; *p* values derived from logistic regression models; Trend test: χ^2^ test for a linear trend in the odds ratios; BLQ = below limit of quantification.

## Data Availability

The dataset used in this study will be made available to qualified researchers upon reasonable requests to the corresponding author, subject to institutional data use agreements and ethical approvals.

## Research in context

### Evidence before this study

Experimental studies show that pesticides such as glyphosate, 2,4-D, and cypermethrin cause hepatotoxicity through oxidative stress, mitochondrial dysfunction, and fibrogenic pathways. Epidemiological evidence remains limited and often relies on occupational or crop-type proxies prone to misclassification. Within TIGER-LC, we previously demonstrated that urinary glyphosate and its metabolites were elevated in patients with CLD and HCC compared with hospital-based controls, implicating pesticide exposure as a risk factor but restricted to single-compound analyses without cumulative or predictive modelling.

### Added value of this study

We extended urinary profiling to eight additional pesticides and derived a composite PLS to capture cumulative internal burden. This score was incorporated into two predictive models—a logistic regression–based PILCRS and an XGBoost classifier—across 593 TIGER-LC participants. Both demonstrated strong discrimination and calibration, with PILCRS achieving an optimism-corrected AUC of 0·91. CLD probability increased from 30% in the lowest quartile to more than 70% in the highest. Independent associations for cypermethrin and glyphosate metabolites were retained after adjustment for demographics and lifestyle. Sensitivity analyses confirmed robustness across subgroups, and calibration plots indicated close agreement between predicted and observed risks.

### Implications of all the available evidence

This study reframes pesticide burden as a modifiable determinant of liver disease, shifting from single-compound toxicology to cumulative, predictive stratification. The MLRP framework enables scalable surveillance and early prevention, adaptable to AI-enabled public health tools. Findings align with WHO–FAO priorities and support SDG 3·9, underscoring the urgency of regulation, biomonitoring, and protection for vulnerable populations in LMICs.

## Abbreviations

2,4-D: 2,4-dichlorophenoxyacetic acid
AUC: area under the receiver operating characteristic curve
AI: artificial intelligence
AMPA: aminomethylphosphonic acid
BMI: body mass index
CI: confidence interval
CLD: chronic liver disease
DNA: deoxyribonucleic acid
FAO: Food and Agriculture Organization
FC: fold-change
GC–MS: gas chromatography–mass spectrometry
HBC: hospital-based controls
HBV: hepatitis B virus
HCC: hepatocellular carcinoma
HCV: hepatitis C virus
L1: Lasso regularisation (penalises the absolute magnitude of coefficients, enabling feature selection)
L2: Ridge regularisation (penalises the squared magnitude of coefficients, retaining all features)
LC–MS/MS: liquid chromatography–tandem mass spectrometry
LMICs: low- and middle-income countries
LOD: limit of detection
LOQ: limit of quantification
MLRP: machine learning risk prediction
OR: odds ratio
PILCRS: Pesticide-Informed Liver Cancer Risk Score
PILCRS_8_: PLS_8_-based score with clinical covariates
PILCRS_11_: PLS_11_-based score with clinical covariates
PILCRS_CYP_: cypermethrin-based score with clinical covariates
PLS: Pesticide Load Score
PLS_8_: PLS based on eight urinary pesticide analytes
PLS_8_ FC: fold-change in PLS_8_, normalised to the control group median
PLS_11_: PLS based on eleven urinary analytes, including glyphosate and its metabolites (AMPA, PPA)
PLS_11_ FC: fold-change in PLS_11_, normalised to the control group median
PPA: phosphoric acid
ROC: receiver operating characteristic
SD: standard deviation
SDG: Sustainable Development Goal
SHAP: Shapley Additive Explanations
STROBE: Strengthening the Reporting of Observational Studies in Epidemiology
TIGER-LC: Thailand Initiative in Genomics and Expression Research for Liver Cancer
TRIPOD: Transparent Reporting of a Multivariable Prediction Model for Individual Prognosis or Diagnosis
TQ-S: triple quadrupole–sensitive
UPLC: ultra-performance liquid chromatography
WHO: World Health Organization
XGBoost: Extreme Gradient Boosting

## References

[1] Organization WH. Report of the 17th FAO/WHO Joint Meeting on Pesticide Management; 2024.

[2] Cavalier H, Trasande L, Porta M. Exposures to pesticides and risk of cancer: Evaluation of recent epidemiological evidence in humans and paths forward. Int J Cancer 2023; 152(5): 879–912. 36134639 10.1002/ijc.34300 PMC9880902

[R3] IAfRoC. Agents Classified by the IARC Monographs, Volumes 1–125. 2019 https://monographs.iarc.who.int/agents-classified-by-the-iarc/ (accessed July 18 2025).

[4] Landrigan PJ, Fuller R, Acosta NJR, The Lancet Commission on pollution and health. Lancet 2018; 391(10119): 462–512. 29056410 10.1016/S0140-6736(17)32345-0

[5] Nations U. Goal 3: Ensure healthy lives and promote well-being for all at all ages. 2015. https://sdgs.un.org/goals/goal3 (accessed July 18 2025).

[6] Kuwata K, Inoue K, Ichimura R, Takahashi M, Kodama Y, Yoshida M. Constitutive active/androstane receptor, peroxisome proliferator-activated receptor alpha, and cytotoxicity are involved in oxadiazon-induced liver tumor development in mice. Food Chem Toxicol 2016; 88: 75–86. 26710982 10.1016/j.fct.2015.12.017

[7] Ahmad MI, Zafeer MF, Javed M, Ahmad M. Pendimethalin-induced oxidative stress, DNA damage and activation of anti-inflammatory and apoptotic markers in male rats. Sci Rep 2018; 8(1): 17139. 30459330 10.1038/s41598-018-35484-3 PMC6244357

[8] Taha MAI, Badawy MEI, Abdel-Razik RK, Younis HM, Abo-El-Saad MM. Mitochondrial dysfunction and oxidative stress in liver of male albino rats after exposing to sub-chronic intoxication of chlorpyrifos, cypermethrin, and imidacloprid. Pestic Biochem Physiol 2021; 178: 104938. 34446205 10.1016/j.pestbp.2021.104938

[9] Patel DP, Loffredo CA, Pupacdi B, Associations of chronic liver disease and liver cancer with glyphosate and its metabolites in Thailand. Int J Cancer 2025; 156(10): 1885–97. 39653658 10.1002/ijc.35282 PMC11924304

[10] Seven B, Kultigin, Cavusoglu, Yalcin E, Acar A. Investigation of cypermethrin toxicity in Swiss albino mice with physiological, genetic and biochemical approaches. Sci Rep 2022; 12(1): 11439. 35794216 10.1038/s41598-022-15800-8 PMC9259609

[11] Martins RX, Carvalho M, Maia ME, 2,4-D Herbicide-Induced Hepatotoxicity: Unveiling Disrupted Liver Functions and Associated Biomarkers. Toxics 2024; 12(1). 10.3390/toxics12010035 PMC10818579 38250991

[12] VoPham T, Bertrand KA, Hart JE, Pesticide exposure and liver cancer: a review. Cancer Causes Control 2017; 28(3): 177–90. 28194594 10.1007/s10552-017-0854-6 PMC5336347

[13] Agency USEP. Cypermethrin; Pesticide Tolerances. Federal Register; 2025.

[14] Pupacdi B, Loffredo CA, Budhu A, The landscape of etiological patterns of hepatocellular carcinoma and intrahepatic cholangiocarcinoma in Thailand. Int J Cancer 2024; 155(8): 1387–99. 38761410 10.1002/ijc.35034 PMC11326978

[15] Suljevic D, Ibragic S, Mitrasinovic-Brulic M, Focak M. Evaluating the effects of anticoagulant rodenticide bromadiolone in Wistar rats co-exposed to vitamin K: impact on blood-liver axis and brain oxidative status. Mol Cell Biochem 2022; 477(2): 525–36. 34816338 10.1007/s11010-021-04303-1

[16] Yang B, Liu Y, Li Y, Exposure to the herbicide butachlor activates hepatic stress signals and disturbs lipid metabolism in mice. Chemosphere 2021; 283: 131226. 34146870 10.1016/j.chemosphere.2021.131226

[17] Coronado-Posada N, Mercado-Camargo J, Olivero-Verbel J. In Silico Analysis to Identify Molecular Targets for Chemicals of Concern: The Case Study of Flocoumafen, an Anticoagulant Pesticide. Environ Toxicol Chem 2021; 40(7): 2034–43. 33729601 10.1002/etc.5042

[18] Mie A, Ruden C, Grandjean P. Safety of Safety Evaluation of Pesticides: developmental neurotoxicity of chlorpyrifos and chlorpyrifos-methyl. Environ Health 2018; 17(1): 77. 30442131 10.1186/s12940-018-0421-y PMC6238321

[19] Samanta P, Bandyopadhyay N, Pal S, Mukherjee AK, Ghosh AR. Histopathological and ultramicroscopical changes in gill, liver and kidney of Anabas testudineus (Bloch) after chronic intoxication of almix (metsulfuron methyl 10.1%+chlorimuron ethyl 10.1%) herbicide. Ecotoxicol Environ Saf 2015; 122: 360–7. 26318971 10.1016/j.ecoenv.2015.08.022

[20] Panuwet P, Siriwong W, Prapamontol T, Agricultural Pesticide Management in Thailand: Situation and Population Health Risk. Environ Sci Policy 2012; 17: 72–81. 22308095 10.1016/j.envsci.2011.12.005 PMC3269779

[21] Charatcharoenwitthaya P, Karaketklang K, Aekplakorn W. Impact of metabolic phenotype and alcohol consumption on mortality risk in metabolic dysfunction-associated fatty liver disease: a population-based cohort study. Sci Rep 2024; 14(1): 12663. 38830939 10.1038/s41598-024-63453-6 PMC11148152

[22] Laohaudomchok W, Nankongnab N, Siriruttanapruk S, Pesticide use in Thailand: Current situation, health risks, and gaps in research and policy. Hum Ecol Risk Assess 2021; 27(5): 1147–69. 34290491 10.1080/10807039.2020.1808777 PMC8291370

[23] Liu Y, Wu F. Global burden of aflatoxin-induced hepatocellular carcinoma: a risk assessment. Environ Health Perspect 2010; 118(6): 818–24. 20172840 10.1289/ehp.0901388 PMC2898859

[24] Rizzati V, Briand O, Guillou H, Gamet-Payrastre L. Effects of pesticide mixtures in human and animal models: An update of the recent literature. Chem Biol Interact 2016; 254: 231–46. 27312199 10.1016/j.cbi.2016.06.003

[25] Vermeulen R, Schymanski EL, Barabasi AL, Miller GW. The exposome and health: Where chemistry meets biology. Science 2020; 367(6476): 392–6. 31974245 10.1126/science.aay3164 PMC7227413

[26] Wild CP, Scalbert A, Herceg Z. Measuring the exposome: a powerful basis for evaluating environmental exposures and cancer risk. Environ Mol Mutagen 2013; 54(7): 480–99. 23681765 10.1002/em.21777

[27] Chen TQ, Guestrin C. XGBoost: A Scalable Tree Boosting System. Kdd’16: Proceedings of the 22nd Acm Sigkdd International Conference on Knowledge Discovery and Data Mining 2016: 785–94.

[28] Steyerberg EW, Vickers AJ, Cook NR, Assessing the performance of prediction models: a framework for traditional and novel measures. Epidemiology 2010; 21(1): 128–38. 20010215 10.1097/EDE.0b013e3181c30fb2 PMC3575184

[29] Collins GS, Reitsma JB, Altman DG, Moons KG. Transparent Reporting of a multivariable prediction model for Individual Prognosis or Diagnosis (TRIPOD): the TRIPOD statement. Ann Intern Med 2015; 162(1): 55–63. 25560714 10.7326/M14-0697

[30] von Elm E, Altman DG, Egger M, The Strengthening the Reporting of Observational Studies in Epidemiology (STROBE) statement: guidelines for reporting observational studies. Lancet 2007; 370(9596): 1453–7. 18064739 10.1016/S0140-6736(07)61602-X

