# Supplementary material for: Urinary pesticide profiles and liver disease risk in Thailand: a machine-learning risk-prediction model
Source: medRxiv. 2025 Sep 22:2025.09.19.25336162. Preprint. [Version 1] doi: 10.1101/2025.09.19.25336162 (PMC12754707; doi:10.1101/2025.09.19.25336162)
Supplement: Supplement 1 [file media-1.pdf]

Urinary pesticide profiles and liver disease risk in Thailand: a machine-learning risk-prediction model

Daxesh P. Patel, Christopher A. Loffredo, Majda Haznadar, Mohammed Khan, Amelia L. Parker, Benjarath Pupacdi, Siritida Rabibhadana, Panida Navasumrit, Nirush Lertprasertsuke, Anon Chotirosniramit, Chawalit Pairojkul, Vor Luvira, Ake Pugkhem, Wattana Sukeepaisarnjaroen, Teerapat Ungtrakul, Thaniya Sricharunrat, Kannika Phornphutkul, Frank J. Gonzalez, Anuradha Budhu, Chulabhorn Mahidol, Xin W. Wang, Mathuros Ruchirawat, and Curtis C. Harris; TIGER-LC Consortium

Targeted LC–MS/MS Assay for Cross-Sectional Quantification of Urinary Pesticides: Sample Preparation, Instrument Parameters, and Analytical Performance

Urine samples were processed using a reversed-phase extraction protocol optimised for small-molecule pesticide detection. Briefly, 60 µL of urine was diluted 1:5 with extraction buffer (acetonitrile:water:methanol, 65:30:5, v/v) containing chlorpropamide (2 µM) as an internal standard. After centrifugation at 15 000 × g for 15 min, 250 µL of supernatant was transferred to a 96-well plate and diluted 1:2 with 50% methanol.

Target compounds were quantified using LC–MS/MS on a XEVO-TQSmicro triple quadrupole mass spectrometer (Waters Corporation, USA) with electrospray ionisation in both positive and negative modes. Chromatographic separation was performed on an ACQUITY UPLC BEH C18 column (1.7 µm, 50 × 2.1 mm) maintained at 40 °C, with a 5 µL injection volume. Source parameters included a capillary voltage of 2.39 kV, source temperature of 150 °C, desolvation temperature of 500 °C, and desolvation gas flow of 1000 L/h. Data acquisition was performed in multiple reaction monitoring (MRM) mode with transitions optimised using IntelliStart.

The following precursor–product ion transitions (m/z), cone voltages (V), and collision energies (eV) were used: pendimethalin, 282.11 → 212.04 (16, 8); oxadiazon, 345.04 → 219.97 (42, 18); metsulfuron-methyl, 141.10 → 42.68 (72, 18); butachlor, 312.13 → 238.05 (18, 10); 2,4-dichlorophenoxyacetic acid (2,4-D), 218.95 → 160.89 (28, 12); cypermethrin, 415.98 → 190.88 (48, 12); flocoumafen, 543.20 → 355.17 (56, 22); and bromadiolone, 525.11 → 250.04 (48, 36). Chlorpropamide (277.07 → 110.96; 28, 30) was used as the internal standard. Quantification was performed using external calibration curves generated from authentic standards in 50% methanol.

Analytical validation showed signal enhancement as the predominant matrix effect across compounds. Limits of detection ranged from 0.16 to 467 nM, and limits of quantification were ≤0.5 nM for six of eight pesticides. Extraction efficiency exceeded 200% across concentration levels, with reproducible recovery and minimal ion suppression. Detection frequencies in the study population ranged from 3.7% (bromadiolone) to 98.6% (cypermethrin), supporting high assay sensitivity under field conditions (Table S1).

Table S1: Targeted LC–MS/MS assay for cross-sectional quantification of urinary pesticides: compound use profiles, mass spectrometry parameters, and analytical performance

ISO common name	Use type	Target pest or weed	Matrix effect	Suppression / Enhancement ratio	LOD (nM)	LOQ (nM)	Extraction efficiency (high, %)	Extraction efficiency (medium, %)	Extraction efficiency (low, %)	Detection frequency (*n* samples)
Pendimethalin	Herbicide	Grasses, broadleaf weeds (microtubule inhibitor)	Enhancement	1·47	0·16	0·5	1810·03	2009·83	270·04	55
Oxadiazon	Herbicide	Grasses, some broadleaves (PPO inhibitor)	Enhancement	1·416	0·5	0·5	412·60	435·90	534·82	66
Metsulfuron-methyl	Herbicide	Broadleaf weeds (ALS inhibitor)	Enhancement	2·14	467	0·5	233·73	328·17	380·89	649
Butachlor	Herbicide	Annual grasses in rice (pre-emergent)	Enhancement	1·416	0·5	13·67	341·29	340·95	395·07	474
2,4-D	Herbicide	Broadleaf weeds (auxin mimic)	Enhancement	1·47	467	467	252·13	240·29	231·23	24
Cypermethrin	Insecticide	Broad-spectrum (pyrethroid)	Enhancement	24	370·3	370·3	354·09	315·57	326·50	1085
Flocoumafen	Rodenticide	Rodents (anticoagulant)	Enhancement	4·48	0·167	4·67	747·32	923·91	938·46	601
Bromadiolone	Rodenticide	Rodents (rats, mice; anticoagulant)	Enhancement	1·438	41	41	280·98	268·80	209·18	22

ISO = International Organization for Standardization. Urine matrix effects predominantly resulted in signal enhancement, indicating that ion suppression was not a major contributor to low detection. All pre-spiked pesticides were efficiently recovered, with extraction inefficiency as the main limiting factor. LOD = lowest concentration with signal-to-noise ratio (SNR) ≥3 (RMS); LOQ = lowest concentration with SNR ≥10 (RMS).

Table S2: Performance characteristics and calibration of internal exposure-based liver disease risk models

Model	Outcome	Corrected AUC	Calibration slope	95% CI (slope)	Calibration interpretation	Thresholds for performance classification
**$PILCRS_{11}$**	CLD	0·890	0·900	0·705–1·118	Excellent	AUC ≥ 0·85 and Slope 0·85–1·15
**$PILCRS_{11}$**	HCC	0·893	0·814	0·617–1·079	Good	AUC ≥ 0·85 and Slope 0·75–1·15
**$PILCRS_{8}$**	CLD	0·834	0·889	0·689–1·110	Good	AUC ≥ 0·80 and Slope 0·85–1·15
**$PILCRS_{8}$**	HCC	0·900	0·840	0·627–1·095	Excellent	AUC ≥ 0·85 and Slope 0·80–1·15
**$PILCRS_{CYP}$**	CLD	0·852	0·772	0·563–1·031	Good	AUC ≥ 0·85 and Slope 0·75–1·15
**$PILCRS_{CYP}$**	HCC	0·904	0·734	0·504–1·060	Acceptable	AUC ≥ 0·85 and Slope ≥ 0·70 with CI crossing 1·0

$PILCRS_{11}$ and $PILCRS_{8}$ denote pesticide-informed liver cancer risk scores derived from logistic regression models incorporating PLS_{11} or PLS_{8} scores and clinical covariates, respectively. $PILCRS_{CYP}$ refers to a cypermethrin-based model. AUC indicates area under the receiver operating characteristic curve; values closer to 1·0 indicate superior discrimination. Calibration slope values near 1·0 indicate good model calibration. 95% CI = 95% confidence interval. Internal validation performed using 1000× bootstrap resamples.

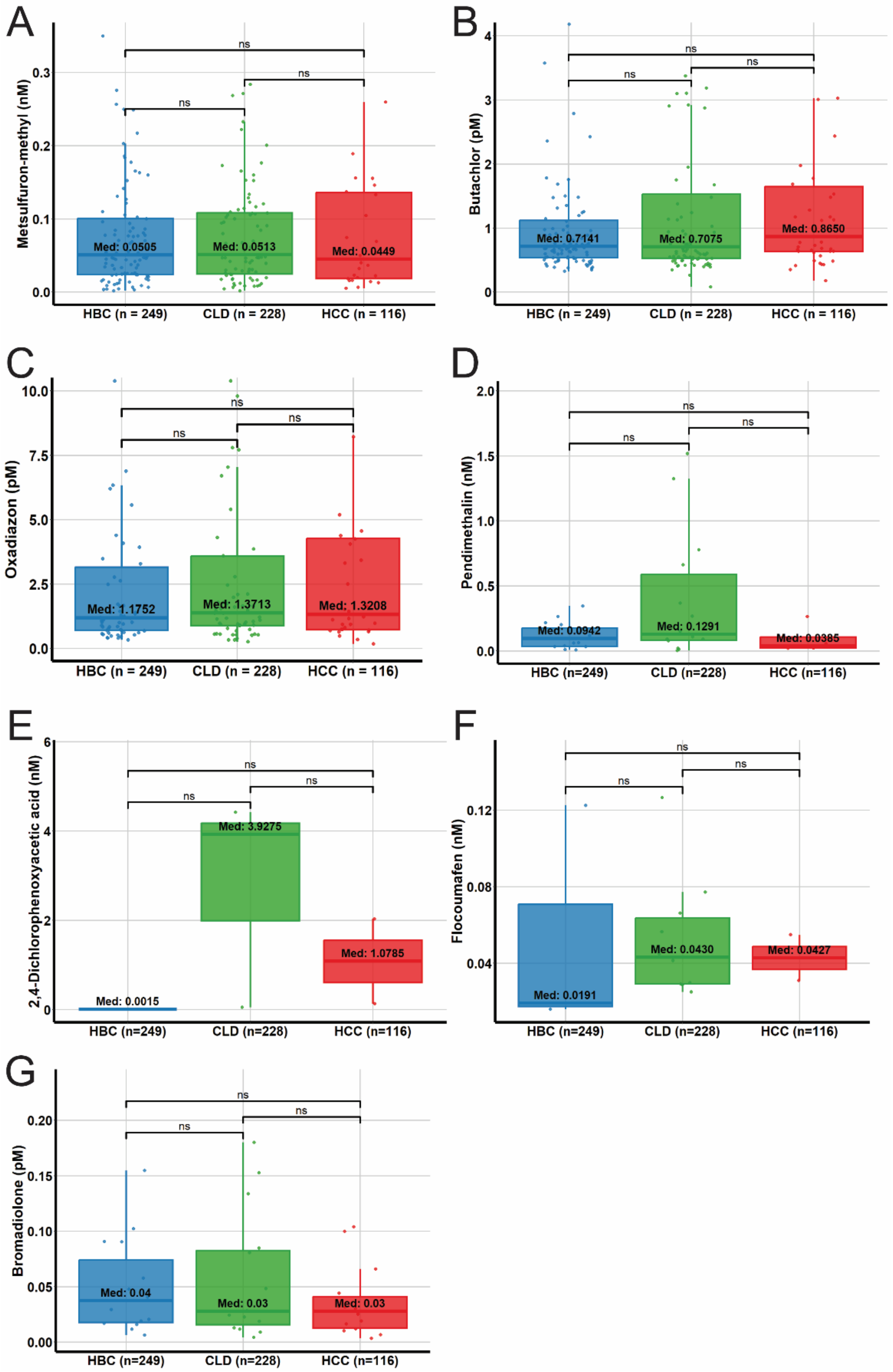

Figure S1: Distribution of urinary pesticide concentrations across disease groups. (A–G) Boxplots show creatinine-adjusted concentrations of seven pesticides—(A) metsulfuron-methyl, (B) butachlor, (C) oxadiazon, (D) pendimethalin, (E) 2,4-D, (F) flocoumafen, and (G) bromadiolone—across HBC (*n* = 249), CLD (*n* = 228), and HCC (*n* = 116). Medians are shown within boxes; horizontal bars denote pairwise comparisons. *ns* = non-significant by Wilcoxon rank-sum test. HBC = hospital-based controls.

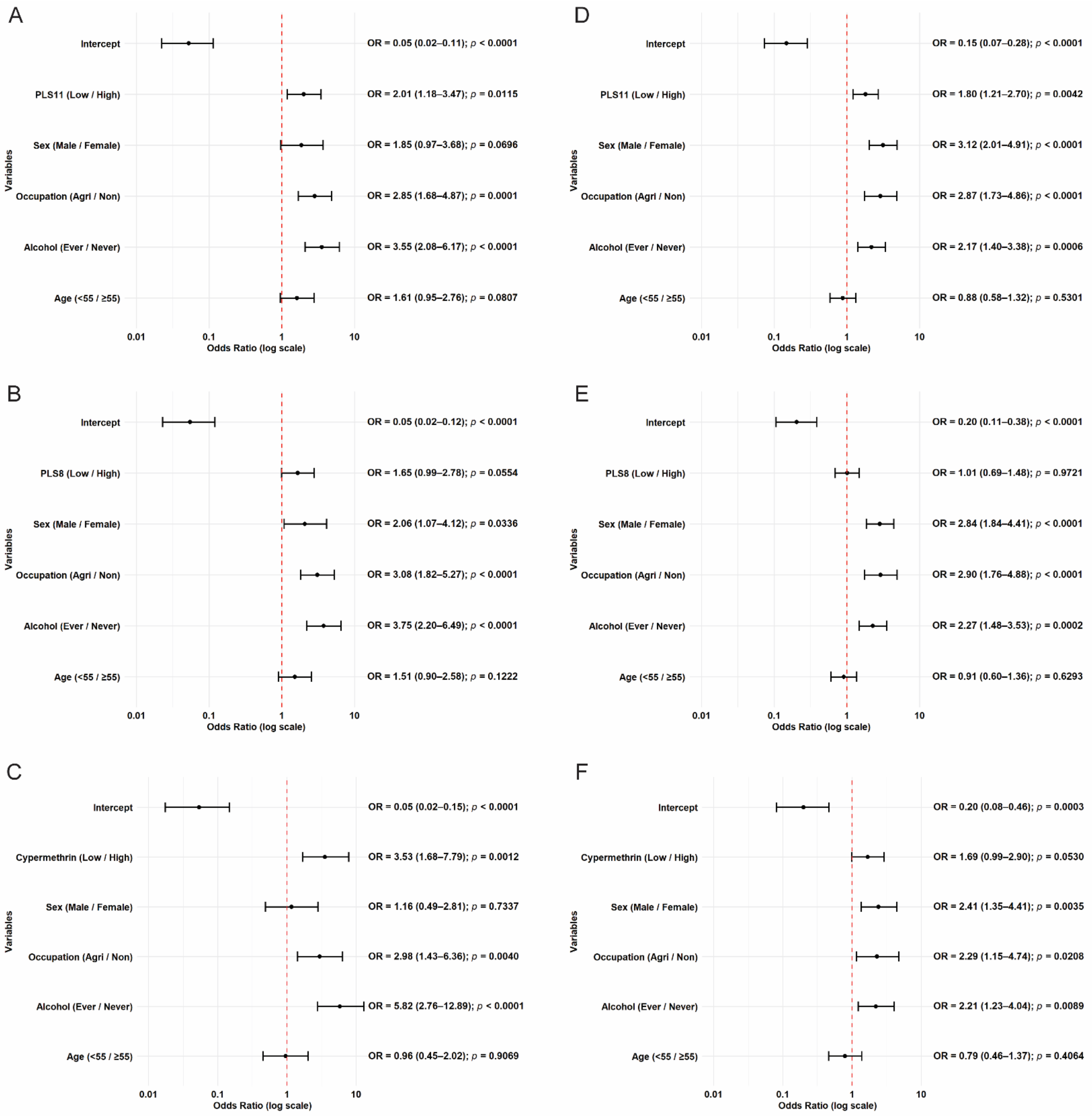

Figure S2: Multivariable-adjusted associations of pesticide exposure and PLS scores with liver disease. (A–C) Forest plots show adjusted odds ratios (ORs, log scale) for CLD versus HBC based on exposure to PLS_{11} (A), PLS_8 (B), and cypermethrin (C), controlling for sex, occupation, alcohol use, and age. (D–F) Corresponding models for HCC versus HBC. Point estimates and 95% confidence intervals are shown; p values are derived from logistic regression models. HBC = hospital-based controls.

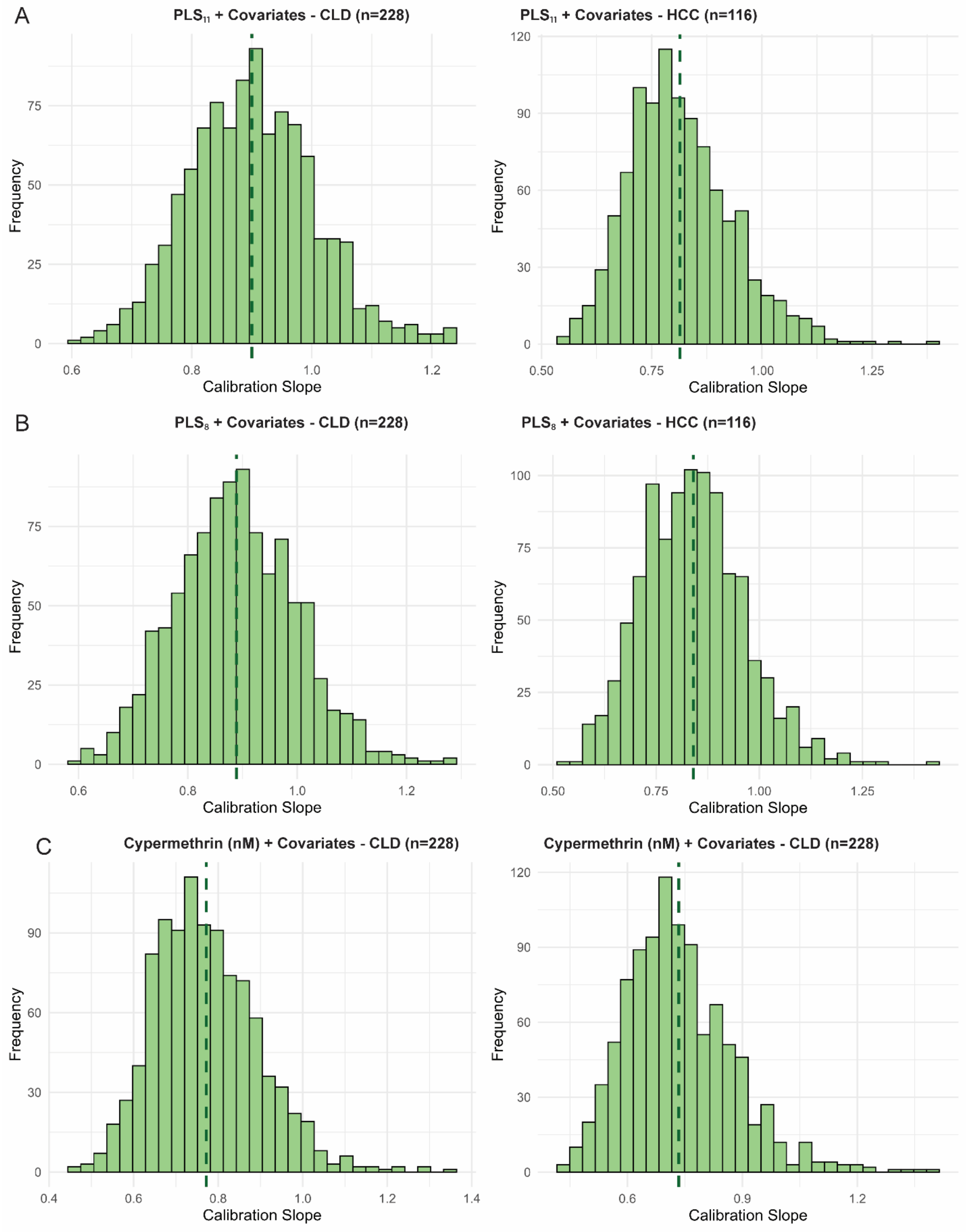

Figure S3: Internal calibration of predictive models for liver disease. (A–C) Histograms show calibration slope distributions from 1000 bootstrap resamples for models predicting CLD (left) and HCC (right) using PLS_{11} (A), PLS_8 (B), and urinary cypermethrin (nM) (C), adjusted for age, sex, occupation, and alcohol use. Dashed lines indicate the median slope; values near 1·0 indicate stronger calibration.

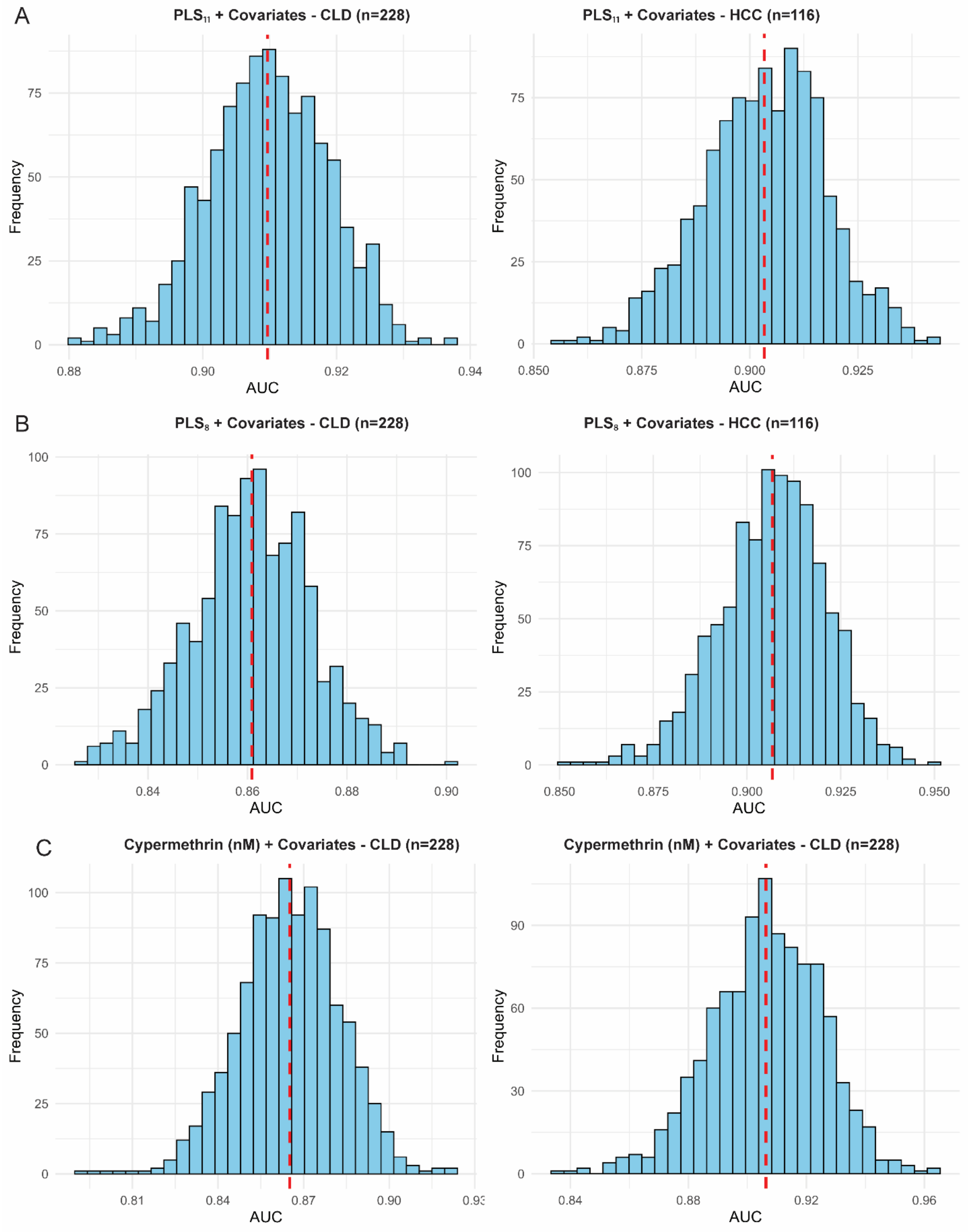

Figure S4: Internal validation of model discrimination. (A–C) Histograms show AUC distributions from 1000 bootstrap resamples for models predicting CLD (left) and HCC (right) using PLS_{11} (A), PLS_8 (B), and urinary cypermethrin (nM) (C), adjusted for age, sex, occupation, and alcohol use. Red dashed lines indicate the median AUC; higher values indicate better discrimination between cases and controls.

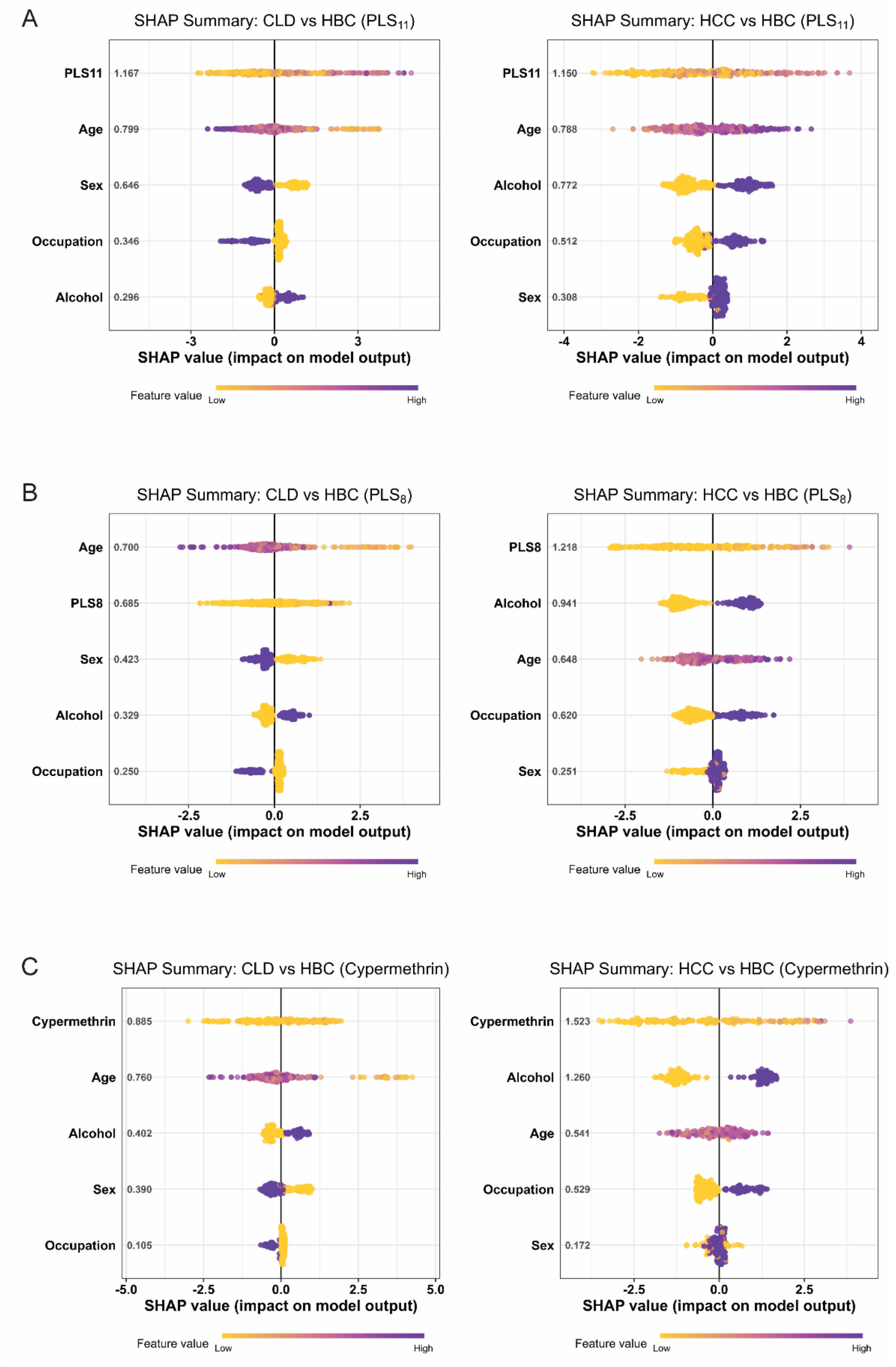

Figure S5: SHAP summary plots for liver disease risk. SHAP plots show feature contributions for CLD (left) and HCC (right) using PLS_{11} (A), PLS_8 (B), and urinary cypermethrin (nM) (C), adjusted for age, sex, occupation, and alcohol use. Features are ranked by mean absolute SHAP value; colour gradients indicate scaled feature expression from low (yellow) to high (purple). Models were trained separately for CLD versus HBC and HCC versus HBC. HBC = hospital-based controls.
